# Challenges in the Provision of Pediatric Palliative Care in Mexico: A Cross-Sectional Web-Based Survey

**DOI:** 10.1177/08258597211062767

**Published:** 2021-12-13

**Authors:** Elena Solveig Grüneberg, Jorge Ramos-Guerrero, Tania Pastrana

**Affiliations:** 1 RWTH Aachen University, Aachen, Germany; 2 Universidad de Guadalajara, Jalisco, Mexico

**Keywords:** health services accessibility, pediatric palliative care, Mexico, children, adolescent, barrier, challenge, low- and middle-income countries

## Abstract

**Objective:** An enormous need for pediatric palliative care (PPC) has been reported, especially in low- and middle-income countries (LMICs). However, the access to PPC is limited. This study identifies the current challenges in the provision of PPC and their severity from the perspective of healthcare professionals. **Method:** We conducted a web-based descriptive cross-sectional survey among healthcare professionals treating children in need of palliative care in Mexico in 2019. We used convenience sampling and snowball sampling to acquire participants. **Results:** Seventy healthcare professionals from Mexico participated. Participants were 64.3% female, on average 45.8 (SD = 10.9) years old, had an average of 15.84 (SD = 10.4) years of work experience and worked in 15 states. The three most severe barriers reported were: *(1) Few teams and/or networks of out-of-hospital/domestic support; (2) Absence of training centres and continuing medical/paramedical education in PPC; and (3) Lack of legal, labor, and economic protection for parents who must stop working to be with their children.* The barriers related to a lack of awareness and commitment, a lack of support, legal factors, and working conditions were rated highest. Participants considered increased awareness and better knowledge of PPC for all as the top priority, and particularly emphasized the need for better education and training of health professionals. **Conclusion:** We have identified several barriers to successful palliative care (PC) provision for children. Primarily, these are lack of awareness and commitment, especially of the health authorities and the medical professions, lack of personal and financial support, legal factors, and working conditions. The need to change and improve care exists at the policy level, the health professional level, and the public societal level.

## Introduction

The International Association for Hospice and Palliative Care defines palliative care (PC) as “the active holistic care of individuals across all ages with serious health-related suffering due to severe illness and especially of those near the end of life. It aims to improve the quality of life of patients, their families and their caregivers”.^1^

Globally, approximately 21 million children need pediatric palliative care (PPC),^2^ most of them in low- and middle-income countries (LMICs).^3^ However, higher levels of PPC provision are available in high-income countries (HICs).^3^ Eliminating the barriers existing in LMICs to access PPC “must become a priority”^4^ and for that, a global intervention is required.^3^

In comparison to adults, children have special PC needs,^5^ since in addition to symptom control, they require developmentally appropriate care—sometimes over years/decades—schooling and care in the family context.^6^ In order to address the needs of young patients and their families, a multidisciplinary team is required.^6^

Mexico is classified by the World Bank^7^ as an (upper) middle-income country and has over 126 million inhabitants (2020), of which approximately 34% are between 0 and 19 years.^8^ In 2010, 546,643 Mexican children needed PPC.^2^ Moreover, Cardenas-Turanzas et al. assumed, “that 80% of deaths of children diagnosed with complex chronic conditions who died [in their study] would have benefited from palliative care”, as well as their legal guardians and relatives.^9^

PPC in Mexico began in the early 2000s with an interdisciplinary in-hospital consultation service provided by the National Institute of Pediatrics in Mexico City.^10^ Although great advances have been made since then,^10^ Mexico reported only six PPC teams (0.17 per million inhabitants under 15 years) in 2020, which are still insufficient to meet the existing need.^11^ Currently, the assessment of the level of development of children's PC in Mexico varies in the literature between “Evidence of capacity building activities for the provision of children's palliative care”.^12^ “Isolated children's palliative care provision” and “Generalised children's palliative care provision”.^3^ In summary, despite the developments of recent years, major challenges remain.^10,13^

The aim of this study was to identify the current challenges in the provision of PPC and their severity from the perspective of healthcare professionals who treat children with life-limiting or life-threatening conditions in Mexico.

## Method

### Design of the Study

We conducted an observational, cross-sectional, web-based open survey to explore barriers to PPC provision from the perspective of healthcare providers who performed PPC in Mexico between January and April 2019.

### Questionnaire

**Figure 1.**
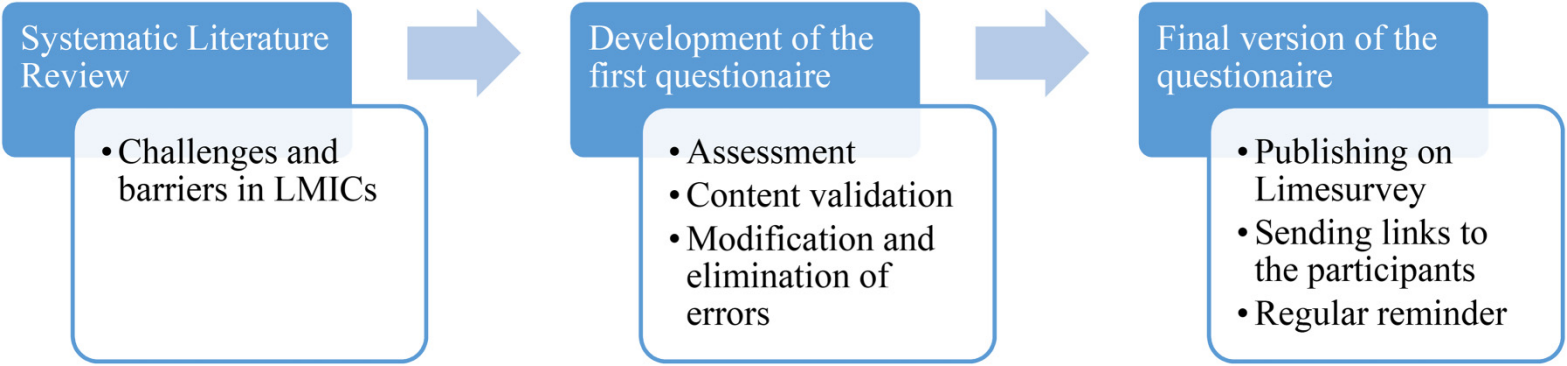
Process description of the development of the questionnaire.

We developed the survey instrument in Spanish based on systematic literature research conducted as part of the underlying dissertation and expert consultation. The instrument was translated for this article and reviewed by native speakers. A group of four experts in PPC and research established content validity and their suggestions were implemented in the final version of the questionnaire (Figure 1)*.* The internal consistency of the barrier subgroups was calculated using Cronbach's alpha.

The questionnaire contained 93 structured questions grouped into three blocks: (1) Sociodemographic background; (2) Professional experience; and (3) Barriers to accessing PPC, which included 73 statements to be rated on a 4-point scale from 0 (no problem) to 3 (serious problem) with the additional option “don't know”. Additionally, we asked two open questions about missing barriers and their own priorities within the development of PPC. At the end of the questionnaire, we gave them the possibility to add comments and to share views and experiences.

The survey was developed using Lime Survey®, provided by the Medical Faculty of the Rheinisch-Westfälische Technische Hochschule (RWTH) Aachen. We distributed an invitation with the survey link via email among potential informants, following up with four reminders. We provided the participants with general information about the study and the objectives of the survey on the first page of the questionnaire. Before responding, they had to confirm that their participation was voluntary and that they were healthcare professionals treating children with severe illnesses. No incentives were offered for their participation. No protection was used to prevent double participation.

### Study Sample

Since there is no register of healthcare workers providing PPC, we used convenience sampling and snowball sampling. We disseminated the survey through both an informal group of PPC providers and several additional groups/societies, possibly including healthcare professionals working with pediatric patients with PC needs. We further asked participants to share the questionnaire with other potential candidates. The study only included healthcare professionals who answered “yes” to the mandatory question at the beginning asking whether they treat children with PC needs.

### Statistical Analysis

We stored the data in a secure account on the server at RWTH Aachen, Germany. For the statistical analysis, we exported the results into the Statistical Package for Social Sciences (IBM SPSS 26). We cleared all data sets that did not provide answers to all mandatory questions (“incomplete data set”) and conducted a descriptive analysis.

Since the sociodemographic and clinical characteristics of the participants were not normally distributed, the median and the first and third quartiles (Q1-Q3) were given. Since the median did not allow a precise ranking, in some cases the mean was also given.

We conducted a contingency analysis using the Wilcoxon–Mann–Whitney-U-Test (WMW), considering *P* < .05 statistically significant. We examined seven groups for statistically significant differences based on the following characteristics: “age”, “gender”, “work experience”, “degree in paediatrics”, “training in PC”, “education in PPC”, and “working in a team”. For the analysis, we dichotomized age into <45 versus ≥45 years and years of experience into ≤5 years “junior” versus >5 years “senior”. For further analysis of the results of the WMW test the WMW_odds_ were calculated.^14^

We used Microsoft Excel (2016) to thematically analyze the answers to the open questions in Spanish. These were translated to English for this paper.

The ethical approval for this study is registered in the Comité de Ética en Investigación, Hospital Valentín Gómez Farías, ISSSTE under the number 619/2108 ISSSTE/CEI/317/2018.

## Results

The participation rate was 67% (*76 of 113*) and the completion rate was 92%.^15^ Seventy questionnaires were filled out by healthcare professionals, who identified themselves as PPC providers.

Forty-five (64.3%) of the participants were female. The participants were between 30 and 70 years old (*Mdn* *=* *45, Q1-3* *=* *37-54.3*), working in a total of 14 of the 32 states in Mexico. Forty percent of the participants lived and worked in the country's capital, followed by other major cities (Table 1).

**Table 1.** Sociodemographic and Work-Related Information About the Survey Participants (n = 70).

Characteristics	n (%)
Gender	
Women	45 (64.3)
Men	25 (35.7)
Age	
Mdn (Q1-3)	45 (37-54, 25)
State (Cities) of work	
Mexico City	28 (40.0)
Guanajuato (León)	7 (10.0)
Nuevo Leon (Monterrey)	7 (10.0)
Jalisco (Guadalajara, Zapopan)	6 (8.6)
Mexico (Toluca, Ixtapaluca, Tlalnepantla)	4 (5.7)
Baja California (La Paz, Tijuana)	2 (2.9)
Chihuahua (Chihuahua, Ciudad Juarez)	2 (2.9)
Coahuila (Satillo, Torreón)	2 (2.9)
Puebla (Puebla)	2 (2.9)
Queretaro (Queretaro)	2 (2,9)
Tabasco (Villahermosa)	2 (2,9)
Aguascalientes (Aguascalientes)	1 (1.4)
Veracruz (Minatitlán)	1 (1.4)
Yucatán (Mérida)	1 (1.4)
No data	3 (4,3)
Profession	
Physician	
Pediatrician	54
No Pediatrician	9
Other	
Nurse	3
Social Worker	3
Tanatologist	1
Work experience (years)	
Mdn (Q1-3)	15 (6-25) Range: 2 to 39
Training in PC	13 (18.6)
Work setting	
Hospital	50 (71.4)
First Level (**“**practice” and **“**home care”)	2 (2.9)
Both	15 (21.4)
No data	3 (4.3)
Working as/with a team	
Yes	26 (37.1)
No	44 (62.9)

Abbreviation: PC: palliativecare.

More than two-thirds of the participants were pediatricians (*n* *=* *54; 77%),* of them 94% (*n* *=* *51*) had an additional specialization in neuropediatrics (*n* *=* *17*), oncology (*n* *=* *15*), hematology (*n* *=* *12*), or nephrology (*n* *=* *1*). The remaining (*n* *=* *6*) did not report their specialization.

The participants had a median of 15 years of work experience (*Q1-3* *=* *6-25*). Most participants (n = 50; 71%) worked exclusively in hospitals, 3% exclusively at the first level of care provision (“*practice*” and “*home care*”) and 21% worked in both settings.

Thirteen (*18.6%*) participants reported some training in PC and six of them (*8.6%*) stated to have specific training in PPC. The remaining participants did not report any kind of training in PC/PPC (Table 1).

The proportion of children in the total number of patients varied between 5% and 100% (*Mdn* *=* *87.5%; Q1-3* *=* *20-100*), while 46.4% (*n* *=* *26*) exclusively treated pediatric patients. All in all, 7.1% of participants treated exclusively children with PPC needs, 50% treated them frequently and 42.9% sometimes. The participants estimated to have attended to 2 to 20 000 children the previous year (*Mdn* *=* *80, Q1-3* *=* *25-200*), 1 to 580 children (*Mdn* *=* *15, Q1-3* *=* *5-30*) had PC needs and 0 to 200 children died while in their care (*Mdn* *=* *5, Q1-3* *=* *2-10*). The reported treatment period varied from hours to 8 years. (Table 2)

**Table 2.** Patients Characteristics.

Characteristics	
Children dedication	
<80%	n = 27 (48.2%)
≥ 80%	n = 29 (51.8%)
Children attended /year (Sum = 42** **078)	Mdn = 80 (Q1-3 = 25-200) Range: 2 to 20 000
Children with PPC need/year (Sum = 3056)	Mdn = 15 (Q1-3 = 5-30) Range: 1 to 580
Treatment frequency of children with PPC needs	Exclusive n = 5 (7.1%) Frequently n = 35 (50%) Sometimes n = 30 (42.9%)
Deceased children/ year (Sum = 802)	Mdn = 5 (Q1-3 = 2-10) Range: 0 to 200
Treatment period	From a few hours to many years

Participants reported that most patients receiving PPC had oncological or hematological conditions, but also diseases of the nervous system, congenital malformations and conditions related to the perinatal period (Table 3).

**Table 3.** Pathologies (ICD 10).

Oncologic	C00-D49 Neoplasms *(eg, C22.2 Hepatoblastoma, C30.0 Malignant neoplasm of adrenal gland, C41 Malignant neoplasm of bone and articular cartilage, Other Sarcoma, C64 Malignant neoplasm of kidney, C69.2 Malignant neoplasm of retina, C71 Malignant neoplasm of brain, C72 Malignant neoplasm of spinal cord, cranial nerves and other parts of central nervous system, C74 Malignant neoplasm of nasal cavity, C79.4 Secondary malignant neoplasm of other and unspecified parts of nervous system, C81-C96 Malignant neoplasms of lymphoid, hematopoietic and related tissue)* D60-D64 Aplastic and other anemias and other bone marrow failure syndromes D70 Neutropenia *(eg, D70.1 Agranulocytosis secondary to cancer chemotherapy,)*
Non-Oncologic	A00-B99 Certain infectious and parasitic diseases (*eg A15-A19 Tuberculosis, A41 Other Sepsis, A87 Viral meningitis)* D50-D89 Diseases of the blood and blood-forming organs and certain disorders involving the immune mechanism *(eg, D55-D59 Hemolytic anemias, D59.3 Hemolytic-uremic syndrome, D76.1 Hemophagocytic lymphohistiocytosis)* E00-E89 Endocrine, nutritional and metabolic diseases *(eg, E40-E46 Malnutrition, E71.52 Adrenoleukodystrophy, E75 Disorders of sphingolipid metabolism and other disorders of lipid storage, E77.0 Defects in post-translational modification of lysosomal enzymes, E84.0 Cystic fibrosis with pulmonary manifestations)* F01-F99 Mental, Behavioral and Neurodevelopmental disorders *(eg, F84 Pervasive developmental disorders)* G00-G99 Diseases of the nervous system *(eg G00 Bacterial meningitis, G40 Epilepsy, G71.0 Muscular dystrophy, G80 Infantile cerebral palsy)* I00-I99 Diseases of the circulatory system *(eg, I26-I28 Pulmonary heart disease and diseases of pulmonary circulation, I33.0 Acute and subacute infective endocarditis, I40.0 Infective myocarditis, I42 Cardiomyopathy, I42.0 Dilated cardiomyopathy, I67 Other cerebrovascular diseases)* J00-J99 Diseases of the respiratory system *(eg, J10 Influenza, J69 Pneumonitis due to solids and liquids)* K00-K95 Diseases of the digestive system *(eg, K63 Other diseases of the intestine, K72.0 Acute and subacute hepatic failure)* M00-M99 Diseases of the musculoskeletal system and connective tissue *(eg M32 Systemic lupus erythematosus [SLE])* N17-N19 Acute kidney failure and chronic kidney disease P00-P96 Certain conditions originating in the perinatal period *(eg, P07 Disorders related to short pregnancy and low birth weight, P84 Asphyxia prenatal)* Q00-Q99 Congenital malformations, deformities, and chromosomal abnormalities *(eg, Q00.0 Anencephaly, Q21.3 Tetralogy of Fallot, Q24 Other congenital malformations of heart, Q33.3 Agenesis of lung, Q44.2 Atresia of bile ducts, Q93 Monosomies and deletions from the autosomes, not elsewhere classified)* S00-T88 Injury, poisoning and certain other consequences of external causes *(eg S00-S09 Head injuries, T07 Unspecified multiple injuries, T81 Complications of procedures)*

### Barriers to Accessing Palliative Care

Ninety-nine percent of all barriers listed in the questionnaire (n = 72 of 73) were rated as at least “Moderate” in median (Mdn>2). The highest ranked barrier by mean was “Few teams and/or networks of out-of-hospital/domestic support” (*M* *=* *2.66, SD* *=* *0.57*) and the one most frequently rated as serious was “Lack of legal, labour, and economic/financial protection to protect parents who must stop working to be with their children” (*74.5% of all valid answers*). All barriers inquired about in the questionnaire can be found in the Appendix ( *Supplemental Material 1* ).

**Figure 2.**
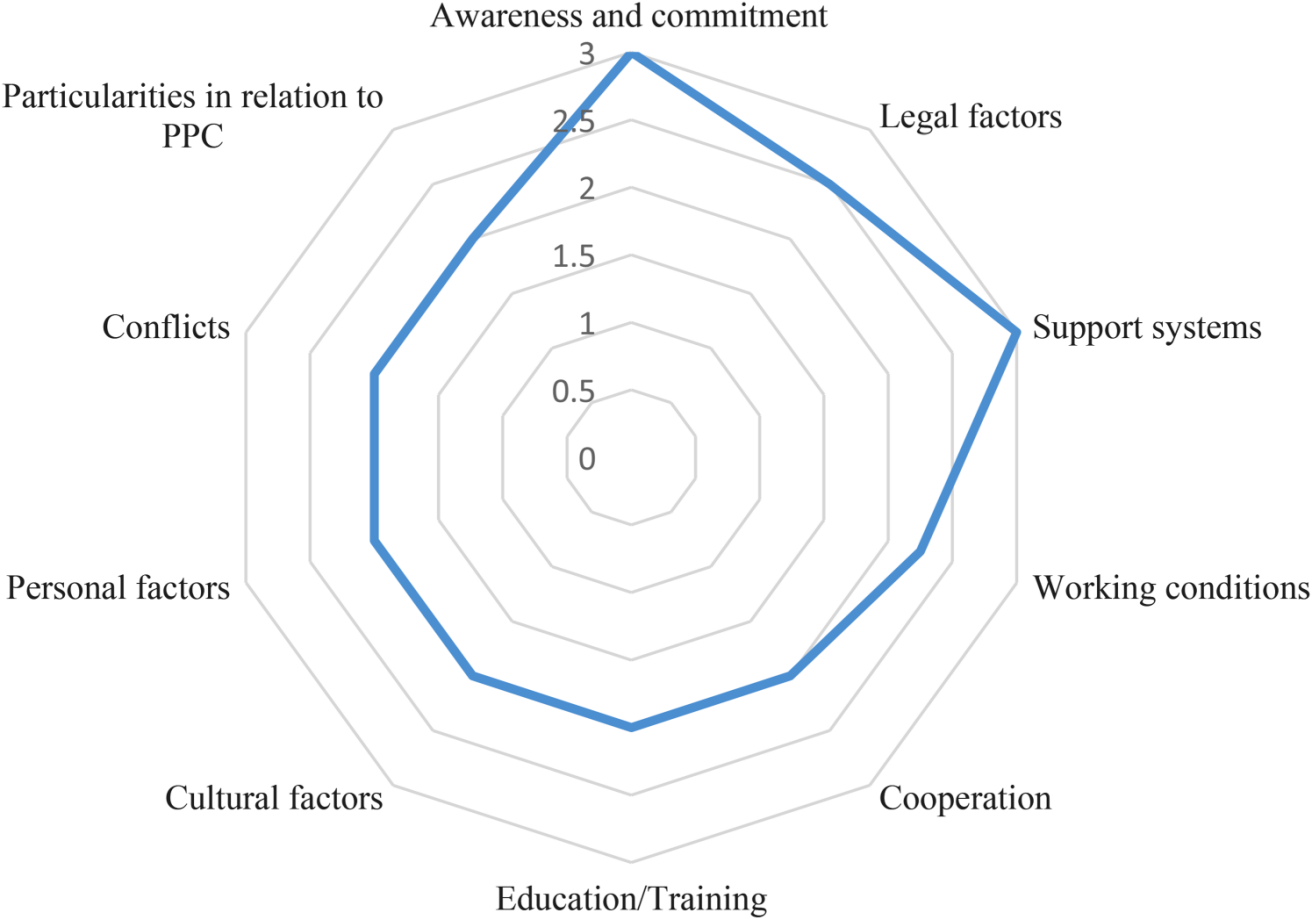
Illustration of the distribution of the subgroups according to the median.

The 73 barriers statements were categorized in 10 subgroups according to the contents (Figure 2). The internal consistency of the individual subgroups was acceptable to excellent (*Cronbach's Alpha: .7-.9*). The subgroup that referred to the right awareness and commitment, eg from politicians, health authorities, or the medical profession (*Mdn* *=* *3, Q1-3* *=* *2.25-3*) and the subgroup that referred to support systems, eg economic, local/ambulatory, for primary caregivers/parents/patients or health professionals (*Mdn* *=* *3, Q1-3* *=* *2.25-3*) were rated highest. Followed by the subgroups related to legal factors (*Mdn* *=* *2.5, Q1-3* *=* *2-3*) and working conditions (eg staff, geographical distribution, location, time) (*Mdn* *=* *2.25, Q1-3* *=* *2-3*). The remaining six subgroups had the same median (*Mdn* *=* *2*).

The 19 barriers, which were rated as severe by at least 50% of the participants (*Mdn* *=* *3*), described barriers from 7 of the 10 subgroups, namely “Support” (*n* *=* *4 of 6;* Lack of PPC teams/networks in inpatient and outpatient care and financial issues), “Awareness and Commitment” (*n* *=* *4 of 6;* Low commitment of health authorities and the medical profession, lack of budget and absence of a national programme to promote PPC), “Education and Training” (*n* *=* *3 of 12;* Lack of knowledge about PPC and lack of trainers/training opportunities), “Legal factors” (*n* *=* *2 of 4;* “*Gaps between existing legislation and its implementation”*, “*Unclear legal situation regarding adequacy of treatment and advance directives”*), “Working conditions” (*n* *=* *3 of 8;* “*Fragmentation of service provision between institutions”,* “*Lack of assigned and trained staff”,* “*Palliative care teams are concentrated in urban related hospitals.”)*, “Personal factors” (*n* *=* *2 of 13;* “*Work overload”* and “*Prejudice or lack of knowledge about palliative care in the general population.”*), and “Cooperation”(*n* *=* *1 of 7;* “*Difficulties with patient referral and back-referral between different services and levels of care.”*) (Table 4).

**Table 4.** Barriers with a Median of 3.

Barrier	Subgroups
Few teams and/or networks of out-of-hospital/domestic support	Support
Lack of legal, labor, and economic protection for parents who must stop working to be with their children	
Poverty and unfavorable socioeconomic situation	
Absence of a pediatric palliative care team at the site where you work	
Absence of training centres and continuing medical/paramedical education in pediatric palliative care	Education/ Training
Lack of trained teachers to educate the health team on pediatric palliative care	
Lack of knowledge about palliative care among health personnel	
Gaps between existing legislation and its implementation	Legal factors
Unclear legal situation regarding adequacy of treatment and advance directives	
Fragmentation of service provision between institutions	Working conditions
Lack of assigned and trained staff	
Palliative care teams are concentrated in urban-related hospitals	
Little commitment from health authorities to advance pediatric palliative care	Awareness/ Commitment
Lack of budget for pediatric palliative care	
Absence of a national pediatric palliative care program	
Little commitment from the medical professions to advance pediatric palliative care	
Work overload	Personal factors
Prejudice or lack of knowledge about palliative care in the general population	
Difficulties with patient referral and back-referral between different services and levels of care	Cooperation

Results of the contingency analysis of the statements as well as the sociodemographic and educational/professional features of the participants are shown in Table 5. The differentiation based on the factor “Working in a team” revealed statistically significant differences for 19 barriers in all subgroups except for the subgroup “Particularities in relation to PPC”. The chance of a randomly selected participant from the team group rating one of the 19 barriers as less severe was 1.95 to 4.49 to 1.

**Table 5.** Difference in the Assessment of the Barriers According to Sociodemographic and Professional Characteristics by Statements Where *P-*Value was Less Than 5%.

Barrier/Groups (Subgroups)	WMW test (*P* exact)	WMW test (U)	WMW test (Z)	Group 1 (n)/Group 2 (n)	M_rank_ G1/G2	WMW_odds_ G1:G2
Age				<45 years/≥45years	<45 years/≥45 years	<45 years: ≥45 years
Insufficient time to care for patients with palliative needs (Working conditions)	.035	180.5	−2.159	26/21	20.44/28.44	2.02:1
Education in PC				No/Yes	No/Yes	No:Yes
Lack of essential medicines for palliative care, particularly opioids, in presentations suitable for children/in child-friendly forms (Medication/Supplies)	.022	91	−2.266	39/9	21.89/32.89	2.86:1
Education in PPC				No/Yes	No/Yes	No:Yes
Conflict among family members about treatment goals (Conflicts)	.023	31.5	−2.195	41/4	24.23/10.38	1:4.21
Difficulty defining roles among professionals (eg curative vs palliative; pain medicine vs palliative medicine) (Education/Training)	.038	29.5	−2.098	38/4	22.72/9.88	1:4.15
Working in a team				No	No/Yes	No:Yes
Bureaucratic difficulties (forms, recipes, etc.) (Legal factors)	<.001	95	−3.908	29/18	29.72/14.78	1:4.49
Absence of an ethics committee to support decision-making (Support)	<.001	107	−3.525	29/18	29.31/15.44	1:3.88
Absence of a national pediatric palliative care program (Awareness and Commitment)	.001	113	−3.281	27/17	26.81/15.65	1:3.06
Absence of a pediatric palliative care team at the site where you work (Support)	.002	132	−3.087	29/18	28.45/16.83	1:2.95
Insufficient time to care for patients with palliative needs (Working conditions)	.003	139	−2.912	29/18	28.21/17.22	1:2.76
Problems interacting with other patient care services (Cooperation)	.006	141	−2.785	29/18	28.14/17.33	1:2.70
Unrealistic expectations of parents regarding the disease (Personal factors)	.004	134	−2.929	27/18	27.04/16.94	1:2.63
Interests that are not based on the child's welfare (Conflicts)	.007	138	−2.702	27/18	26.89/17.17	1:2.52
Social stigma of a child dying at home (Cultural factors)	.015	130	−2.410	26/17	25.5/16.65	1:2.4
Lack of adequate physical space for care (Working conditions)	.013	157	−2.454	29/18	27.59/18.22	1:2.32
Little commitment from health authorities to advance pediatric palliative care care (Awareness and Commitment)	.005	176	−2.749	29/20	28.93/19.3	1:2.30
Lack of training in the management of emotional stress generated by the treatment of children with pediatric palliative care needs (Education/Training)	.020	143	−2.320	26/18	26/17.44	1:2.27
Lack of trained teachers to educate the health team on pediatric palliative care (Education/Training)	.015	154.5	−2421	27/18	26.28/18.08	1:2.15
Difficulties with patient referral and back-referral between different services and levels of care (Cooperation)	.020	157	−2.303	29/17	26.59/18.24	1:2.14
Lack of knowledge or training in emotional support (Education/Training)	.043	154	−2.021	26/18	25.58/18.06	1:2.04
Difficulty defining roles among professionals (eg curative vs palliative; pain medicine vs palliative medicine) (Education/ Training)	.049	142	−1.980	24/18	24.58/17.39	1:2.04
Lack of awareness and interest in the palliative needs of patients and their families (Awareness and Commitment)	.043	161.5	−2.030	27/18	26.02/18.47	1:2.01
Conflict among family members about treatment goals (Conflicts)	.044	162	−2.045	27/18	26/18.5	1:2
Shortage of medicines and medical supplies (Working conditions)	.048	177	−1.958	29/18	26.9/19.33	1:1.95

Abbreviations: PC: palliative care; PPC: pediatric palliative care; WMW: Wilcoxon–Mann–Whitney-U-test.

Statistically significant differences were present also in the barriers “Conflict among family members about treatment goals” and “Difficulty defining roles among professionals (eg curative vs palliative; pain medicine vs palliative medicine)”. They were rated less severe by participants with training in PPC than by participants without training with a chance of 4.21 to 1 and 4.15 to 1. Additionally, the barrier “Lack of essential medicines for PC, particularly opioids, in presentations suitable for children/in child-friendly forms” was rated less severe by participants without PC training than by their colleagues with training, with a chance of 2.86 to 1. Furthermore, the barrier “Insufficient time to care for palliative patients” was rated as less severe by younger participants (<45 years) with a chance of 2.02 to 1.

As additional barriers, participants mentioned high treatment costs, lack of knowledge about the existence of PPC (“*Lack of knowledge that this branch of medicine exists and that it is part of medical practice.”*), lack of psychological/psychiatric support for the family, and the lack of a 24-h emergency number for caregivers who are caring for patients alone at home.

### Areas of Priority

PPC is seen by the informants as an urgent need in the country:

A palliative care team is required in all paediatric care centers where not only children with cancer but also premature babies are seen. (Paediatrican)

The focus of the participants in their prioritization was on creating the right awareness of the pressing issue and the need to spread knowledge about the principles of PC.

The main priority is palliative care education for health personnel, and in this way the population will be educated, and children will be able to receive palliative care with adequate instruction from health personnel and the family. (Anaesthetist/No Paediatrician)

Disseminating knowledge is also considered important to reduce the fears and debunk myths among the population. This is especially important for all those directly affected:

Work on the dissemination and training of personnel on palliative care, its scope and objectives in order to destigmatise the concepts of death and the care of patients with chronic or terminal illnesses. (Paediatrician)

Participants mentioned the need to engage more politicians and people in charge to be able to build necessary programs and define norms and standards for the implementation of services, especially for more legal and financial support:

Involve the government in including this type of programs by law, with budget and planning. (Paediatrician)

Establish criteria and timely management of Palliative care (No Paediatrician/ No medical specialty indicated)

Three additional aspects to improve timely access to PPC services were also particularly important, namely better dissemination of PPC services (“*Dissemination of Paediatric Palliative Care Centers Providing Paediatric Palliative”*, formation of trained PPC teams *(*“*The formation of specific palliative teams for paediatrics”;* “*Creation of institutional multidisciplinary groups”;* “*Most of the time palliative care is also provided by oncologists and that wears us down more, not only because of the workload but also in the issue”*), and improving access to medications and training.

## Discussion

We assessed the barriers in the provision of PPC from the perspective of 70 healthcare professionals and their severity. Seventy-two of 73 barriers were perceived as moderate or severe. The barrier rated highest on average was “*Few teams and/or networks of out-of-hospital/domestic support”* and the barrier rated by most participants as serious (*n* *=* *35 of 47, 74%)* was “*Lack of legal, labour, and economic protection for parents who must stop working to be with their children”*. Both barriers demonstrate the difficult situation for parents who must take care of a seriously ill child—mostly without sufficient financial and personal support. Besides the lack of economic protection for caregivers, poverty, and a lack of budget for PPC were mentioned as other important financial barriers. The relevance of financial barriers is also reflected by the frequent mention in the international literature published to date on PPC in LMIC.^16‐19^ Eden et al.^20^ reported on the high treatment dropout rate in LMICs due to the costs of therapy, travel, and accommodation for caregivers.

Most of the barriers were structural and organizational which request more action of responsible political players and clinic administrations. It would be particularly important, according to participants’ comments, to show more commitment to raising awareness of PPC among all people, to establish a good legal/regulatory basis, to provide an adequate budget, to build a national PPC program, and to provide support. Torres Vigil et al.^21^^(p323)^ reported that in Mexico “Palliative care [is] not a priority in [the] formulation of healthcare policy”, which among other aspects is reflected in legally highly restricted access to opioids and the lack of prioritization in education.

In our results, the barrier “Difficulties with regulation of opioid prescription” was considered equally as a serious (*n* *=* *14, 20%*), moderate (*n* *=* *16, 22.9%*) and minor barrier (*n* *=* *14, 20%*). This could be explained by the different socioeconomic statuses of the individual states,^22^ despite policy changes such as the simplification of the opioids prescription process in recent years.^23^ Further research on the impact of the different health and social security institutions and the current changes, like the end of the “Seguro Popular” and the creation of the “Instituto de Salud para el Bienestar” (INSABI), would be supportive at this point.

Education accounts for another large share of the barriers rated above average. This area splits into three interrelated areas: the lack of basic knowledge about PPC among the population and the professional sector, the lack of trainers/training opportunities, and the lack of sufficiently qualified personnel. Additionally, only a small percentage of the participants who stated that they treat terminally ill children had received any kind of formal training in PC and even a smaller amount of the participants received a specific training in PPC. The lack of knowledge, training and experience is also one of the frequently mentioned barriers in the international literature about PPC in LMICs.^16,19,24‐27^ Connor and Sisimayi^16^ called these barriers as the “key human resource gaps”. The exploration of ways to raise awareness of PPC in the clinic and in the outpatient setting among health professionals, leaders, especially hospital managers and policy makers, as well as the general population would be useful.

PPC is not yet recognized as a medical specialty. Since 2011, only one training center for PPC “Curso de Alta Especialidad” exists at the National Paediatric Institute and the National Autonomous University of Mexico, where 19 PPC specialists have been trained (1-year residency). Furthermore, out of 33 universities with more than 160 programs offering pediatric training, we found that only two pediatric training programs (at the Universidad de Guadalajara and the Monterrey Institute of Technology and Higher Education) have included PPC in their core curriculum.

The fact that working in a team significantly reduced the perception of the severity of 26% of the barriers queried in our study may have several reasons. The presence of a PPC team already shows a higher awareness and knowledge of PPC at these sites and thus some barriers are less present (eg “*Lack of awareness and interest in the palliative needs of patients and their families.”,* “*Little commitment from health authorities to advance pediatric palliative care care”*). Furthermore, the work as well as the responsibility in a team could be shared among all, so that the individual burden could be experienced as less of a pressure (cf “*Insufficient time to care for patients with palliative needs”,* “*Lack of training in the management of emotional stress generated by the treatment of children with paediatric palliative care needs.”*). Based on this result, further investigation of the influence of working in a team would be useful.

The most frequently reported diagnoses of patients receiving PPC were oncological diseases, especially leukemia and lymphoma. These are also the most frequent cancer types in children, according to country data.^28,29^ This may suggest a work overload for oncologists due to insufficient time for care responsibilities and the emotional burden caused by the lack of training in PPC*.* The close connection between PPC and cancer is probably related with the common myth that PPC is only for cancer patients^30^ as well as the history of PPC in Mexico, which was initiated from “outpatient pain clinics that were unable to meet the complex needs of advanced cancer patients” in the late 1980s and 1990s^21^. However, the internationally literature indicates that the neonatal group is the one with the biggest need.^31^

## Limitations

Due to the ratio between the number of participants and the number of items, it was not possible to conduct a principal component analysis (PCA).

We do not know the total number of professionals offering PPC. We reached a small sample that is likely to be more interested in PPC and that is probably not representative for the whole country. The main criterion used to select participants was the answer to the mandatory question at the beginning, which asked whether the participant was caring for children with PC needs. The exclusion of all participants who answered no to this question is a probable additional reason for the low number of participants.

The number of participants, who choose not to answer all questions, increased toward the end of the questionnaire. This may be due to the length of the questionnaire, which is why we would recommend shortening it if it is used again.

## Conclusion

A series of barriers impede the access to PC for children in Mexico. They related in particular to the lack of awareness and commitment, especially of the health authorities and the medical professions, the lack of personal and financial support, legal factors, and working conditions. Apart from that, the participants prioritized creating awareness and disseminating knowledge. Further research into the individual barriers mentioned above, as well as exploring ways to raise awareness of PPC in clinical and outpatient settings among health professionals, people in positions of responsibility, and the general population, would be useful.

Furthermore, our analysis pointed to a particularly positive relevance of the team factor, so that derived from this, further studies on the effects of working in a team would also be useful.

## Supplemental Material

sj-docx-1-pal-10.1177_08258597211062767 - Supplemental material for Challenges in the Provision of Pediatric Palliative Care in Mexico: A Cross-Sectional Web-Based Survey Click here for additional data file. Supplemental material, sj-docx-1-pal-10.1177_08258597211062767 for Challenges in the Provision of Pediatric Palliative Care in Mexico: A Cross-Sectional Web-Based Survey by Elena Solveig Grüneberg, Jorge Ramos-Guerrero, and Tania Pastrana in Journal of Palliative Care
