# Supplementary material for: Challenges in the Provision of Pediatric Palliative Care in Mexico: A Cross-Sectional Web-Based Survey
Source: J Palliat Care. 2021 Dec 13;39(1):58–67. doi: 10.1177/08258597211062767 (PMC10687805; doi:10.1177/08258597211062767)
Supplement: sj-docx-1-pal-10.1177_08258597211062767 - Supplemental material for Challenges in the Provision of Pediatric Palliative Care in Mexico: A Cross-Sectional Web-Based Survey [file sj-docx-1-pal-10.1177_08258597211062767.docx]

Supplement 1: All barriers inquired about the questionnaire

| Barriers | M (SD) | MDN (Q1-3) | Serious Barrier n (%) | Moderate Barrier  n (%) | Minor Barrier n (%) | No Barrier n (%) | Do not know & No Answer n (%) |
| --- | --- | --- | --- | --- | --- | --- | --- |
| Awareness and commitment (e.g. politicians, health authorities, medical professions) | 2.34 | 3 |  |  |  |  |  |
| Little commitment from health authorities to advance paediatric palliative care | 2.49 (0.79) | 3 (2-3) | 32 (45.7) | 10 (14.3) | 6 (8.6) | 1 (1.4) | 21 (30) |
| Lack of budget for paediatric palliative care | 2.46 (0.81) | 3 (2-3) | 28 (40.0) | 13 (18.6) | 3 (4.3) | 2 (2.9) | 24 (34.2) |
| Absence of a national paediatric palliative care program | 2.43 (0.87 | 3 (2-3) | 28 (40.0) | 9 (12.9) | 5 (7.1) | 2 (2.9) | 26 (37.1) |
| Little commitment from the medical professions to advance paediatric palliative care | 2.30 (0.86 | 3 (2-3) | 26 (37.1) | 15 (21.4) | 7 (10.0) | 2 (7.0) | 20 (28.5) |
| Lack of awareness and interest in the palliative needs of patients and their families | 2.18 (0.91) | 2 (2-3) | 20 (28.6) | 16 (22.9) | 6 (8.6) | 3 (4.3) | 25 (35.7) |
| The different institutions of the National Health System do not pay attention to PPC | 2.16 (0.9) | 2 (2-3) | 19 (27.1) | 17 (24.3) | 6 (8.6) | 3 (4.3) | 25 (35.7) |
| Support systems (Economical, Local/Outpatient, for primary caregivers/parents/patients, health care professionals) | 2.33 | 3 |  |  |  |  |  |
| Few teams and/or networks of out-of-hospital/domestic support | 2.66 (0.57) | 3 (2-3) | 31 (44.3) | 11 (15.7) | 2 (2.9) | 0 (0.0) | 26 (37.1) |
| Lack of legal, labour, and economic protection for parents who must stop working to be with their children | 2.62 (0.74) | 3 (2-3) | 35 (50.0) | 7 (10.0) | 4 (5.7) | 1 (1.4) | 23 (32.8) |
| Poverty and unfavourable socio-economic situation | 2.54 (0.71) | 3 (2-3) | 32 (45.7) | 14 (20.0) | 3 (4.3) | 1 (1.4) | 20 (28.5) |
| Absence of a paediatric palliative care team at the site where you work | 2.15 (1.08) | 3 (1-3) | 25 (35.7) | 10 (14.3) | 6 (8.6) | 6 (8.6) | 23 (32.8) |
| Lack of support for primary caregivers | 2.13 (0.94) | 2 (2-3) | 19 (27.1) | 17 (24.3) | 5 (7.1) | 4 (5.7) | 25 (35.7) |
| Absence of an ethics committee to support decision-making | 1.89 (1.03) | 2 (1-3) | 17 (24.3) | 13 (18.6) | 12 (17.1) | 5 (7.1) | 23 (32.8) |
| Legal factors | 2.28 | 2.5 |  |  |  |  |  |
| Gaps between existing legislation and its implementation | 2.57 (0.66) | 3 (2-3) | 28 (40.0) | 14 (20.0) | 1 (1.4) | 1 (1.4) | 26 (37.1) |
| Unclear legal situation regarding adequacy of treatment and advance directives | 2.53 (0.71) | 3 (2-3) | 31 (44.3) | 14 (20.0) | 3 (4.3) | 1 (1.4) | 21 (30) |
| Bureaucratic difficulties (forms, recipes, etc.) | 2.19 (0.9) | 2 (2-3) | 22 (31.4) | 14 (20.0) | 9 (12.9) | 2 (2.9) | 23 (32.8) |
| Difficulties with regulation of opioid prescription | 1.83 (0.95) | 2 (1-3) | 14 (20.0) | 16 (22.9) | 14 (20.0) | 4 (5.7) | 22 (31.4) |
| Working conditions (e.g. staff, geographical distribution, location, time, medication/supplies) | 2.28 | 2.25 |  |  |  |  |  |
| Fragmentation of service provision between institutions | 2.52 (0.72) | 3 (2-3) | 30 (42.9) | 10 (14.3) | 6 (8.6) | 0 (0) | 24 (34.2) |
| Lack of assigned and trained staff | 2.49 (0.8) | 3 (2-3) | 31 (44.3) | 9 (12.9) | 6 (8.6) | 1 (1.4) | 23 (32.8) |
| Palliative care teams are concentrated in urban related hospitals | 2.36 (0.93) | 3 (2-3) | 25 (35.7) | 10 (14.3) | 4 (5.7) | 3 (4.3) | 28 (40) |
| Geographical distribution of the population served | 2.30 (0.84) | 2.5 (2-3) | 25 (35.7) | 17 (24.3) | 6 (8.6) | 2 (2.9) | 20 (28.5) |
| Insufficient time to care for patients with palliative needs | 2.30 (0.81) | 2 (2-3) | 23 (32.9) | 16 (22.9) | 7 (10.0) | 1 (1.4) | 23 (32.8) |
| Lack of adequate physical space for care | 2.21 (0.86) | 2 (2-3) | 21 (30.0) | 17 (24.3) | 7 (10.0) | 2 (2.9) | 23 (32.8) |
| Shortage of medicines and medical supplies | 2.13 (0.9) | 2 (1-3) | 20 (28.6) | 15 (21.4) | 10 (14.3) | 2 (2.9) | 23 (32.8) |
| Lack of essential medicines for palliative care, particularly opioids, in presentations suitable for children/in child-friendly forms | 1.91 (1.06) | 2 (1-3) | 18 (25.7) | 13 (18.6) | 10 (14.3) | 6 (8.6) | 23 (32.8) |
| Education and Training | 2.13 | 2 |  |  |  |  |  |
| Absence of training centres and continuing medical/paramedical education in paediatric palliative care | 2.63 ( 0.66 | 3 (2-3) | 31 (44.3) | 8 (11.4) | 4 (5.7) | 0 (0) | 27 (38.6) |
| Lack of trained teachers to educate the health team on paediatric palliative care | 2.51 ( 0.76 | 3 (2-3) | 29 (41.4) | 11 (15.7) | 4 (5.7) | 1 (1.4) | 25 (35.7) |
| Lack of knowledge about palliative care among health personnel | 2.39 (0.84) | 3 (2-3) | 25 (35.7) | 13 (18.6) | 4 (5.7) | 2 (2.9) | 26 (37.1) |
| Poor training of health personnel in management and leadership skills | 2.24 (0.91) | 2 (2-3) | 22 (31.4) | 15 (21.4) | 5 (7.1) | 3 (4.3) | 25 (35.7) |
| Lack of knowledge or training in grief support/ bereavement support | 2.16 (0.96) | 2 (2-3) | 20 (28.6) | 15 (21.4) | 5 (7.1) | 4 (5.7) | 26 (37.1) |
| Lack of training in the management of emotional stress generated by the treatment of children with paediatric palliative care needs | 2.11 (0.97) | 2 (2-3) | 19 (27.1) | 15 (21.4) | 6 (8.6) | 4 (5.7) | 26 (37.1) |
| Lack of knowledge or training in spiritual support | 2.11 (0.99) | 2 (1.25-3) | 20 (28.6) | 13 (18.6) | 7 (10.0) | 4 (5.7) | 26 (37.1) |
| Lack of knowledge or training in emotional support | 2.05 (0.99) | 2 (1-3) | 18 (25.7) | 14 (20.0) | 8 (11.4) | 4 (5.7) | 26 (37.1) |
| Lack of knowledge or training in communication in difficult situations | 2.00 (1.00) | 2 (1-3) | 18 (25.7) | 13 (18.6) | 10 (14.3) | 4 (5.7) | 25 (35.7) |
| Difficulty in defining roles among professionals (e.g. curative vs. Palliative; pain medicine vs. palliative medicine) | 1.98 (0.92) | 2 (1-3) | 15 (21.4) | 13 (18.6) | 12 (17.1) | 2 (2.9) | 28 (40) |
| Lack of training in pain management | 1.82 (1.15) | 2 (1-3) | 17 (24.3) | 10 (14.3) | 9 (12.9) | 8 (11.4) | 26 (37,1) |
| Lack of training in managing other symptoms | 1.64 (1.12) | 2 (1-3) | 13 (18.6) | 11 (15.7) | 11 (15.7) | 9 (12.9) | 26 (37.1) |
| Personal factors (prejudices, emotional difficulties, expectations, insecurities) | 2.09 | 2 |  |  |  |  |  |
| Work overload | 2.44 (0.81) | 3 (2-3) | 28 (40) | 10 (14.3) | 6 (8.6) | 1 (1.4) | 25 (35.7) |
| Prejudice or lack of knowledge about palliative care in the general population | 2.43 (0.73) | 3 (2-3) | 24 (34.3) | 16 (22.9) | 3 (4.3) | 1 (1.4) | 26 (37.1) |
| Unrealistic expectations of parents regarding the disease | 2.38 (0.53) | 2 (2-3) | 18 (25.7) | 26 (37.1) | 1 (1.4) | 0 (0) | 25 (35.7) |
| Family difficulty in accepting the condition that the child is out of curative treatment | 2.22 (0.77) | 2 (2-3) | 17 (24.3) | 23 (32.9) | 3 (4.3) | 2 (2.9) | 25 (35.7) |
| Difficulties in adapting the patient/family to the disease process of the disease | 2.20 (0.73) | 2 (2-3) | 16 (22.9) | 23 (32.9) | 5 (7.1) | 1 (1.4) | 25 (35.7) |
| Fear/unawareness of adequacy of therapeutic effort at the end of life on the part of the patient and/or family | 2.09 (0.93) | 2 (2-3) | 17 (24.3) | 19 (27.1) | 5 (7.1) | 4 (5.7) | 25 (35.7) |
| Fear or unawareness of the adequacy of end-of-life treatment on the part of health care personnel | 2.07 (1.04) | 2 (1-3) | 20 (28.6) | 12 (17.1) | 7 (10.0) | 5 (7.1) | 26 (37.1) |
| Association of Palliative Care with patient surrender/abandonment (Patient/Family) | 1.98 (0.94) | 2 (1-3) | 14 (20.0) | 18 (25.7) | 7 (10.0) | 4 (5.7) | 27 (38.6) |
| Myths about opioids. e.g. fear of opioid dependence | 1.95 (0.89) | 2 (1-3) | 13 (18.6) | 19 (27.1) | 9 (12.9) | 3 (4.3) | 26 (37.1) |
| Insecurity in dealing with the family and the patient in relation to palliative care | 1.93 (0.94) | 2 (1-3) | 14 (20.0) | 18 (25.7) | 9 (12.9) | 4 (5.7) | 25 (35.7) |
| Difficulty facing the death of children | 1.91 (1.04) | 2 (1-3) | 17 (24.3) | 12 (17.1) | 11 (15.7) | 5 (7.1) | 25 (35.7) |
| Association of ‘Palliative Care’ with surrender or abandonment of the patient (Health care professionals) | 1.88 (1.02) | 2 (1-3) | 15 (21.4) | 11 (15.7) | 12 (17.1) | 4 (5.7) | 28 (40) |
| Fear of opioid dependence | 1.70 (1.11) | 2 (1-3) | 13 (18.6) | 14 (20.0) | 8 (11.4) | 9 (12.9) | 26 (37.1) |
| Cultural factors | 1.95 | 2 |  |  |  |  |  |
| Resistance to talk about "death" | 2.16 ( 0.8 | 2 (2-3) | 17 (24.3) | 19 (27.1) | 8 (11.4) | 1 (1.4) | 25 (35.7) |
| Social stigma of a child dying at home | 2.12 (0.93) | 2 (1-3) | 19 (27.1) | 12 (17.1) | 10 (14.3) | 2 (2.9) | 27 (38.6) |
| Resistance to accepting palliative care (axiom of care vs. cure) | 2.02 (0.99) | 2 (1-3) | 18 (25.7) | 14 (20.0) | 9 (12.9) | 4 (5.7) | 25 (35.7) |
| Phobic medical culture towards death and the failure of curative treatment | 1.89 (1.03) | 2 (1-3) | 16 (22.9) | 13 (18.6) | 11 (15.7) | 5 (7.1) | 25 (35.7) |
| Religious and/or cultural values or beliefs that affect the adequacy of end-of-life treatment | 1.84 (0.80) | 2 (1-2) | 10 (14.3) | 19 (27.1) | 15 (21.4) | 1 (1.4) | 25 (35.7) |
| Religious and/or cultural limitation to specific treatment options (such as blood transfusion) | 1.82 (0.81) | 2 (1-2) | 9 (12.9) | 21 (30.0) | 13 (18.6) | 2 (2.9) | 25 (35.7) |
| Cultural rejection of palliative care | 1.81 (0.92 | 2 (1-3) | 11 (15.7) | 15 (21.4) | 13 (18.6) | 3 (4.3) | 28 (40) |
| Inter-institutional/inter-professional/ interdisciplinary cooperation | 1.91 | 2 |  |  |  |  |  |
| Difficulties with patient referral and back-referral between different services and levels of care | 2.37 (0.88) | 3 (2-3) | 27 (38,6) | 11 (15,7) | 6 (8,6) | 2 (2,9) | 24 (34,3) |
| Problems interacting with other patient care services | 2.09 (0.9) | 2 (1-3) | 19 (27.1) | 15 (21.4) | 11 (15.7) | 2 (2.9) | 23 (32.8) |
| Lack of continuity in patient management | 2.05 (0.99) | 2 (2-3) | 17 (24.3) | 17 (24.3) | 5 (7.1) | 5 (7.1) | 26 (37.1) |
| Difficulty of interaction with other disciplines (therapies, emergencies, oncology) | 1.84 (0.96 | 2 (1-3) | 12 (17.1) | 18 (25.7) | 9 (12.9) | 5 (7.1) | 26 (37.1) |
| Problems in the communication with other teams | 1.77 (0.84) | 2 (1-2) | 10 (14.3) | 14 (20.0) | 18 (25.7) | 1 (1.4) | 27 (38.5) |
| Difficulty working in a team | 1.67 (0.81) | 2 (1-2) | 7 (10.0) | 17 (24.3) | 17 (24.3) | 2 (2.9) | 27 (38.5) |
| Problems in intra-team communication | 1.60 (0.82) | 2 (1-2) | 6 (8.6) | 17 (24.3) | 17 (24.3) | 3 (4.3) | 37 (38.5.4) |
| Conflicts (e.g. communication problems or disagreement about treatment goals) | 1.89 | 2 |  |  |  |  |  |
| Ignorance of children's rights. such as their participation in decisions about their treatment | 2.06 (0.98) | 2 (1-3) | 20 (28.6) | 15 (21.4) | 9 (12.9) | 4 (5.7) | 24 (31.4) |
| Conflict among family members about treatment goals | 2.00 (0.77) | 2 (2-3) | 12 (17.1) | 23 (32.9) | 8 (11.4) | 2 (2.9) | 25 (35.7) |
| Interests that are not based on the child's welfare | 2.00 (0.77) | 2 (2-2.5) | 11 (15.7) | 25 (35.7) | 7 (10.0) | 2 (2.9) | 25 (35.7) |
| Conflicts between staff members and family members about treatment goals | 1.93 (0.82) | 2 (1-2.75) | 11 (15.7) | 21 (30.0) | 10 (14.3) | 2 (2.9) | 26 (37.1) |
| Communication limitations by language (no common language spoken) | 1.87 (0.92) | 2 (1-3) | 13 (18.6) | 16 (22.9) | 13 (18.6) | 3 (4.3) | 25 (35.7) |
| Conflict between staff members about treatment goals | 1.84 (0.92 | 2 (1-3) | 12 (17.1) | 15 (21.4) | 13 (18.6) | 3 (4.3) | 27 (38.5) |
| Communication problems with family/patient requiring palliative care | 1.59 (0.79) | 1.5 (1-2) | 6 (8.6) | 16 (22.9) | 20 (28.6) | 2 (2.9) | 26 (37.1) |
| Particularities in relation to PPC (e.g. identification of need, different stages of development, complex clinical conditions, uncertain prognosis) | 1.87 | 2 |  |  |  |  |  |
| Inherent complexity due to the variability of ages and pathologies | 2.09 (0.80) | 2 (2-3) | 15 (21.4) | 19 (27.1) | 9 (12.9) | 1 (1.4) | 26 (37.1) |
| Uncertainty about the identification of patients requiring paediatric palliative care (timely referral/timely derivation) | 1.91 (1.10) | 2 (1-3) | 18 (25.7) | 12 (17.1) | 8 (11.4) | 7 (10.0) | 25 (35.7) |
| Uncertainty about the prognosis | 1.62 (1.05) | 2 (1-2.5) | 11 (15.7) | 14 (20.0) | 12 (17.1) | 8 (11.4) | 25 (35.7) |
